# Explanatory factors of digital citizenship among university students. A cross-national dataset

**DOI:** 10.1016/j.dib.2024.111011

**Published:** 2024-10-12

**Authors:** Nicoleta Laura Popa, Gabriela Monica Assante

**Affiliations:** Educational Sciences Department, Faculty of Psychology and Educational Sciences, Alexandru Ioan Cuza University of Iași, Iasi, Romania

**Keywords:** Digital citizenship, Self-transcendence values, Civic attitudes, Digital experiences, Youth

## Abstract

The present research explores a set of explanatory factors of digital citizenship in university students on a cross-national sample. The data were collected from 501 university students aged 19–23 years old, from two public universities in Romania (*N* = 350) and Italy (*N* = 151). Information was gathered from one university in each country by using an online survey form. Statistical analyses were conducted using SmartPLS 4.1.0.6. Data analyses included structural equation modelling, mediational analysis, and multigroup analysis. The study focuses on digital citizenship, civic attitudes, personal values, and digital experiences among university students. Rather than making definitive judgments, the goal of this data-driven endeavour is to provide informative insights by identifying patterns within the dataset. The article also describes the stages of data collection and how the modelling of structural equations analysis is used to evaluate the conceptual model of the proposed relationships between variables. In addition to providing information into the current situation, the data discussed in this article is a useful tool for future research and for tailored educational activities, such as digital citizenship training sessions embedded into the academic curricula.

Specifications Table

**Table:** 

Subject	Social science
Specific subject area	Education Citizenship education
Type of data	Primary data, Tables
Data collection	Data were collected using an online survey platform (Google Forms). The questionnaires are previously validated instruments translated using the forward-backward translation method. The collected data was imported into Excel and SPSS and analysed using SmartPLS 4.1 (version 4.1.0.6).
Data source location	The data were collected from students of Alexandru Ioan Cuza University of Iasi, Romania and University of Turin, Italy.
Data accessibility	Data is uploaded on OSF. Repository name: OSF Data identification number: 10.17605/OSF.IO/8RDQE Direct URL to data: https://osf.io/8rdqe/

## 1 Value of the Data

• The data provide empirical evidence about the variability of the core attributes of digital citizenship, according to the country of residence.
• Along with factors that potentially influence digital citizenship (such as personal values, civic attitudes, and digital experiences), the essential component of digital citizenship—i.e., the critical perspective—is discussed.
• The attached dataset provides information gathered in two different cultural contexts, allowing cross-national comparisons.
• The data aspects may support various policymakers in developing sustainable programmes for enhancing university students’ digital citizenship.

## 2 Background

Empirical research on digital citizenship in higher education has grown in popularity in recent years, aligning with a time when technology has spread to every aspect of daily life [[1], [2], [3]]. A recent bibliometric analysis on digital citizenship [4] showed an overlapping with several concepts, including information literacy, empowerment, higher education, primary education, digital literacy, digital competence, and digital citizenship education. However, thorough coverage of digital citizenship within university curricula is likely viewed as a “natural” byproduct of efforts made to enhance students' digital competences and, as such, is often overlooked in policy documents and programme materials. Given the major changes universities have undertaken to better prepare students for future societies, we believe that digital citizenship will further expand the meaning of digital competence development and completely enter the academic debate on transversal competences in higher education. Although the primary goal of the aforementioned education courses is to help students become more proficient in technology use, the publicʼs concern about the threats that young people face online has led to a swift response that focused on internet safety training. This process is more than a means to an end, hence the building blocks of developing digital citizenship must be clarified to develop sustainable programmes. Also, there is evidence that young people's online behaviour depends on their own narratives, stories, and experiences of digital life [5]. However, the results of previous studies show that personal values, such as self-transcendence and security, have a vital role in influencing digital citizen participation [6]. Therefore, deeper understanding of personal values’ influence on digital citizenship could provide direction or create incentive mechanisms and grasp user experiences in digital communities. Further, other studies showed that social factors such as civic attitudes impact youth digital citizenship [7].

**Fig. 1:**
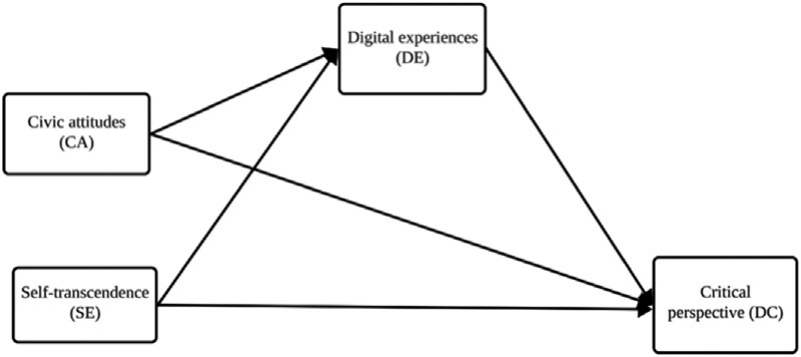
The proposed model.

The data in the present study aim to analyse a set of explanatory factors of digital citizenship, as perceived by university students in two different European cultural contexts. Three dimensions were followed in unfolding predictors for the critical perspective of digital citizenship: self-transcendence individual values, civic attitudes, and digital experiences. Therefore, the data helps explore the values that support digital citizenship, the individual digital experiences that underlie digital citizenship, and the extent of cross-national variations. Self-transcendence values and civic attitudes are thought to have an impact on people's digital experiences, which in turn may have an impact on the critical perspective element of digital citizenship. The data focus on the civic attitudes, the self-transcendence values and the digital experiences that fuse to untangle the digital citizenship-critical perspective. The data collection procedure and the methodological approach for this conceptual model are described the following sections of the paper. This model makes assumptions about the relationships between the variables, and it is evaluated using SmartPLS 4.1. (specific version 4.1.0.6) structural equation modelling (CB-SEM) methodology. The evaluation follows Valerie's [8] guidelines for applying CB-SEM in data articles. The model (Fig. 1) and the collected data are consistently and usefully aligned, highlighting key details such as scales’ attributes and model assessment outcomes.

## 3 Data Description

The analysis is based on a cross-national sample of higher education students from Italy and Romania, and the study's design was used to investigate the role of self-transcendence values, civic attitudes and digital experiences in predicting digital citizenship. The overall culture orientations of West and Eastern Europe are relevant for the present study; thus, West European cultures emphasize egalitarianism, intellectual autonomy and harmony, and value less hierarchy and embeddedness, while East European cultures are characterized by lower egalitarianism and intellectual autonomy, and higher hierarchy. Cultural differences in terms of shared values at societal scale are largely explained by the socioeconomic level, and in turn explain variations of policies in key social domains, such as education, work, and welfare [9]. The research presents a descriptive cross-sectional and cross-national analysis. The sample consisted of 501 university students aged 19 to 23 years old, from two public universities in Romania (*N* = 350) and Italy (*N* = 151). For the present study, convenience sampling was used because the universities were not evenly sampled, and the students answered the questionnaire willingly. Convenience sampling yields the disadvantage of not being representative of the entire population, but it is time- and cost-effective. In this study, it allowed quick primary data collection. We employed Hair et al. [10]'s 10-time recommendation to determine a suitable sample size for the study. By following to this rule, the researchers made sure that the minimum number of survey participants corresponded to or exceeded the total number of paths that lead to a latent variable, which in the present study was 50 (i.e., 5 paths multiplied by 10, including digital experiences as a latent variable). Moreover, 700 undergraduates enrolled in various programs received invitations to participate in this study. The response rate was 71.57 %. According to Hair et al. [11], the complete research sample size (*N* = 501) is deemed suitable for multivariate analysis. The primary criteria for inclusion were that students must have been enrolled in a university study program. No other characteristics or exclusion criteria were employed during the data collection procedure, neither were any based on demographics. The data from Romania was primarily provided by female university students (96 %, *N* = 336). In turn, the data from Italy are more balanced from a gender perspective, with 64.2 % female university students and 33.8 % males. Table 1 shows the sample characteristics. The data can be accessed on the OSF repository [12].

**Table 1:** Participants characteristics.

Sample characteristics	Romania		Italy	
	N	%	N	%
**Gender**				
Female	336	96 %	97	64.2 %
Male	10	2.9 %	51	33.8 %
Not declared	4	1.1 %	3	2.0 %
**Mother's education level**				
Elementary school	2	0.6 %	2	1.3 %
Secondary School	62	17.7 %	47	31.1 %
High-school	228	65.1 %	66	43.7 %
Bachelor level	42	12 %	30	19.9 %
Masters Level or more	16	4.6 %	6	4.0 %
**Father's education level**				
Elementary school	2	0.6 %	4	2.6 %
Secondary School	58	16.6 %	42	27.8 %
High-school	243	69.4 %	73	48.3 %
Bachelor level	32	9.1 %	24	15.9 %
Masters Level or more	15	4.3 %	8	5.3 %
**Students’ field of study**				
Social sciences and economics	351	100 %	137	90.8 %
Humanities	–	–	2	1.3
Life Sciences	–	–	1	0.7 %
Mathematics and Computer sciences	–	–	11	7.3 %

The research was conducted between September 2023 and February 2024. Students were informed about the study in the classroom, and the data was collected using an online form, accessible through a QR code. The very first subsections provided details about the study and the informed consent form. The last section requested the participants’ demographic data. Each answer had to be selected from a list, and forms that contained missing values were unable to be submitted in. Therefore, due to the online form's settings, there were no missing values in the dataset. Using a backward-forward translation technique, two versions of the questionnaire were produced. As such, the questionnaire was administered in Romanian and Italian. The forward-backward translation was performed by a professional translator. The questionnaire was provided in English to qualified translators, who subsequently translated it into Romanian and Italian. Any discrepancies that occurred were discussed and fixed. The questionnaire was divided in five sections, as follows: digital citizenship-critical perspective which includes seven Likert-type items such as ‘*I think online participation is an effective way to make a change to something I believe to be unfair or unjust’,* ranging from 1 (strongly disagree) to 7 (strongly agree) [13,14]; four Likert-type items for self-transcendence value [15], for example ‘*It's very important to him/her to help the people around. He/she wants to care for their well-being’* ranging from 1 (not at all like me) to 6 (very much like me); five civic attitudes items such as *‘I feel responsible for my community’* evaluated on 7-point Likert-type scale (from disagree to agree) [16]; four digital experiences items such as *‘I have regretted some of my posts in digital environments’*, that must be evaluated on a 5-point Likert-type scale (1- never and 5- always) [17], which express a concern regarding online activities; and the demographic section which asked questions referring to students’ field of study, age, country of residence, and parents’ education. The questionnaire is available in the OSF repository [12]. In order to ensure measures’ cross-cultural validity, they were examined for convergent validity and construct reliability based on collected data, by using the Confirmatory Factor Analysis method to determine Composite Reliability (CR), Average Variance Extracted (AVE), and McDonald's omega. Table 2, Table 3 present the results for the complete sample, but also for the Romanian and the Italian samples.

**Table 2:** Reliability and convergent validity.

Construct	Romania			Italy			Complete		
	AVE	CR	McDonald's omega	AVE	CR	McDonald's omega	AVE	CR	McDonald's omega
Self-transcendence value	0.541	0.824	0.647	0.440	0.752	0.642	0.477	0.781	0.682
Civic attitudes	0.498	0.832	0.746	0.559	0.863	0.800	0.518	0.843	0.767
Digital experiences	0.585	0.847	0.779	0.545	0.822	0.708	0.572	0.840	0.763
Digital citizenship	0.430	0.839	0.768	0.394	0.809	0.728	0.416	0.830	0.756

*Note.* The questionnaire is provided as a supplementary file in the OSF repository.

**Table 3:** Items loadings, VIFs and p values.

Construct/items	Romania			Italy			Complete sample		
	Item loading	*p* value	VIF	Item loading	*p* value	VIF	Item loading	*p* value	VIF
Self-transcendence value									
val1	0.614	***	1.332	0.518	**	1.235	0.627	***	1.298
val2	0.540	***	1.367	0.285	**	1.186	0.542	***	1.393
val3	0.794	***	1.320	0.616	***	1.046	0.755	***	1.308
val4	0.553	***	1.635	0.586	***	1.212	0.546	***	1.526
Civic attitudes									
ca1	0.660	***	1.480	0.776	***	1.824	0.706	***	1.569
ca2	0.618	***	1.362	0.721	***	1.717	0.650	***	1.429
ca3	0.682	***	1.461	0.703	***	1.607	0.685	***	1.492
ca4	0.491	***	1.630	0.562	***	1.575	0.511	***	1.634
ca5	0.503	***	1.617	0.515	***	1.494	0.501	***	1.605
Digital experiences									
de1	0.507	***	1.223	0.368	**	1.160	0.446	***	1.210
de2	0.440	***	1.309	0.312	**	2.691	0.417	***	2.383
de3	0.844	***	2.302	0.880	**	2.742	0.857	***	2.379
de4	0.862	***	2.259	0.895	**	1.466	0.872	***	1.244
Digital citizenship									
dc1	0.703	***	1.660	0.697	***	1.603	0.695	***	1.609
dc2	0.528	***	1.319	0.596	***	1.422	0.542	***	1.306
dc3	0.644	***	1.512	0.457	***	1.233	0.599	***	1.411
dc4	0.725	***	1.678	0.740	***	1.713	0.726	***	1.651
dc5	0.493	***	1.261	0.198	n.s.	1.105	0.406	***	1.166
dc6	0.498	***	1.399	0.333	**	1.247	0.464	***	1.315
dc7	0.438	***	1.331	0.633	***	1.466	0.488	***	1.327

*Note:* *** *p* < 0.001; ** *p* < 0.05; n.s. not significant.

The data sets in .xlsx and .sav format, the entire Smart PLS 4.1.0.6 workspace that is exported in a.zip file, the meta-info file that includes the description of the research results of the analysis in .xlsx format, and the research questionnaire in English, as well as a description of the variables and the abbreviations used in the model are available in the OSF repository [12].

## 4 Experimental Design, Materials and Methods

In the present study, structural equation modelling (CB-SEM) was used to examine the proposed research model. One of its benefits is its ability to evaluate complex correlations between multidimensional constructs [18]. It also lowers assumptions about the normal distribution and error terms, which helps address issues associated with small sample sizes [8]. Consequently, the current study's assumptions were tested by CB-SEM employing SmartPLS 4.1. software (specific version 4.1.0.6).

### 4.1 CB-SEM measurement model

The measurement model, often referred to as the outer model, must be closely examined as the first stage in evaluating the validity and reliability of measurement items in PLS. [19,20]. To reach this goal, examination of the measurement models' convergent and discriminant validity must be carried out before moving on to the structural model [18]. The construct reliability was assessed using Composite reliability (CR) and McDonald's omega (ω) reliability index. Convergent validity and construct reliability are shown in Table 2 for the overall sample as well as the country-specific sample. For all constructs, McDonald's omega and CR is higher than the suggested cut-off of 0.65 [21,18]. The factor loadings of every indicator (item) were carefully examined in conjunction with the reliability evaluations. It is recommended that loadings surpass 0.50 [18]. As indicated in Table 3, the standardised factor loading estimates for all items were situated at the lower threshold or exceeded the recommended value, therefore they were deemed acceptable. One item value corresponding to the Italian sample was not significant. The average variance extracted (AVE) was used to evaluate convergent validity, which refers to the agreement of several items measuring the same concept [22]. The average variance extracted (AVE) is a measure that shows the variance captured by a concept in relation to measurement error and must exceed 0.50 to validate the construct for further use [23]. Although general guidelines recommend that AVE values should be above the 0.50 threshold. Fornell and Larcker [24] indicate that lower AVE values can be accepted if composite reliability is higher than 0.6 for suggesting an adequate convergent validity of the construct. Therefore, Table 2 displays AVE results and shows acceptable values, aligning with conventional standards or reaching the lower threshold. Further, the results in the Italian sample show that the third item of the self-transcendence scale has a low factor loading; this might be due to phrasing, resulting in a small percentage of common variance. The third item (i.e., 'It is important to him/her to be loyal to his friends. He/she wants to devote himself to people close to him/her.') refers to close acquaintances, while all the other items refer to the larger groups of people.

### 4.2 Structural equation model

The first stage in evaluating the structural model is to look at multicollinearity using the constructs and the variance inflation factor, or VIF. Values are recommended to be below the 3.3 [23]. As Table 3 shows, all VIFs are below the recommended threshold, therefore the model can be considered free of common method bias (no multicollinearity problems, both for sample countries and the total sample [16] (Table 3). The results show how the civic attitudes (*β* = 0.290; *p* < 0.001) and digital experiences (*β* = 0.137, *p* < 0.050) produce a statistically significant effect on digital citizenship, whereby a higher digital citizenship score corresponds to higher scores for the civic attitudes and digital experiences constructs (Table 4). Self-transcendence values are found insignificant in the complete sample (*β* = 0.097, *p* = 0.129), also in Romania (*β* = 0.069, *p* = 0.439), and Italy (*β* = 0.132, *p* = 0.337). Country specific results are slightly different than the results for the complete sample. Digital experiences (β = 0.101, *t* = 0.799, *p* = 0.425) is not significant in the Italian group while it was found significant in the complete sample (β = 0.137, *t* = 2.446, *p* < 0.001) and the Romanian sample (β = 0.152, *t* = 2.161, *p* < 0.050). In the Italian sample results showed that from the included factors, only civic attitudes (β = 0.334, *t* = 2.642, *p* < 0.050) strongly support the development of digital citizenship. Further, the power of the explanatory model is examined. The SEM based modelling produced an appropriate degree of correction considering that the SMRM = 0.049 and the R^2^ = 0.141 for the endogenous construct digital citizenship in the complete sample. For the Romanian sample, SRMR = 0.058 and the R^2^ = 0.136. The results are slightly higher for the Italian sample, SRMR = 0.066 and the R^2^ = 0.172. The results show relatively low R-squared values for digital citizenship (varying from 0.136 to 0.172), which indicate that other additional factors are involved in developing students' digital citizenship. The differences across the two samples may be explained by taking into account the cultural differences, previously suggested in studies dealing with cultural and personal values. Based on data collected over decades with large national samples, researchers argue that cultural differences in terms of shared values at societal scale are largely explained by the socioeconomic level, and in turn explain variations of policies in key social domains [[25], [26], [27]]. On the other hand, the significant role of digital experiences offered within structured academic programs is supported by studies conducted in diverse cultural settings [28,29], although this factor did not show positive effects for the Italian sample in the present study, and we measured the self-reported level of digital experiences.

**Table 4:** Direct relationships.

Hypotheses	Romania			Italy					Complete			
	B	T	*p*-value	Results	B	T	*p*-value	Results	B	t	*p*-value	Results
H1:SE ->DC	0.069	0.775	0.439	Not supported	0.132	0.962	0.337	Not supported	0.097	1.522	0.129	Not supported
H2:CA ->DC	0.280	2.578	0.010	Supported	0.334	2.642	0.009	Supported	0.290	4.154	0.000	Supported
H3:DE ->DC	0.152	2.161	0.031	Supported	0.101	0.799	0.425	Not supported	0.137	2.445	0.015	Supported
H4:SE ->DE	−0.317	3.338	0.001	Supported	0.014	0.116	0.908	Not supported	−0.253	3.810	0.000	Supported
H5:CA ->DE	0.325	3.660	0.000	Supported	0.022	0.207	0.836	Not supported	0.250	4.100	0.000	Supported
	R-sq				R-sq					R-sq		
DC	0.136				0.172					0.141		
DE	0.209				0.001					0.080		

*Note. B* = Beta Coefficient, *T* = *t* – Statistics, *P* = Probability (P) value.

**Fig. 2:**
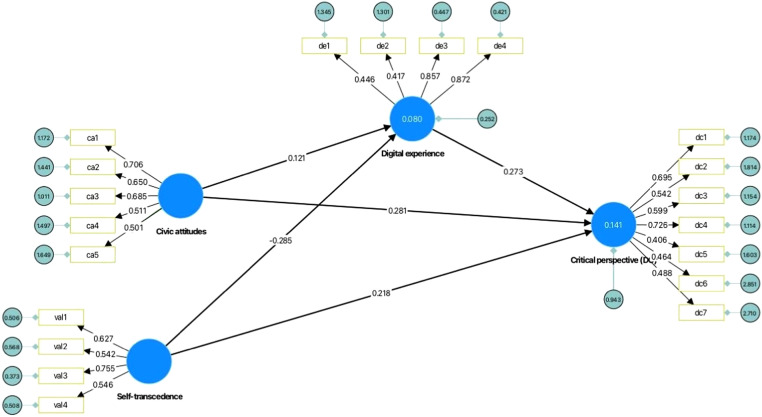
Results of the structural model analysis for the entire sample.

As previous research shows in various cultural contexts, digital competencies have a significant impact on digital citizenship [30], although students display rather basic-intermediate levels of digital competencies at the end of their undergraduate education, but gradually improving along with their study progress [28]. The present study analysed solely the role of digital experiences, civic attitudes and self-transcendence values in digital citizenship development. based on the current results, we can argue that alongside digital competences, civic attitudes and digital experiences would sustain positive citizenship practices in the digital environment. These results may have critical implications for both teacher education and the development of sustainable higher education programmes for digital citizenship enhancement. In line with previous research [31] and given their significance as role-models in students’ academic life, prospective teachers should be actively engaged in programmes promoting he responsible use of digital tools, and fostering collaboration in digital environments. Moreover, similar higher education programmes aiming at digital citizenship development should be made available to all students, given the promising results of studies addressing the effects of such interventions for student teachers [27] (Fig. 2).

### 4.3 Mediation analysis

To evaluate the mediating role of digital experiences in the relationship between civic attitudes and self-transcendence values on digital citizenship, mediation analysis was used. Even though the results showed a negative indirect influence of civic attitudes scores for the entire sample (β = −0.035, *t* = 2.020, *p* = 0.044) and positive indirect influence of self-transcendence values (β = 0.034, *t* = 2.105, *p* = 0.036) on digital citizenship through digital experiences, the results on each country show the opposite. In the Romanian context, the analysis showed different results, an insignificant mediating role of digital experiences from self-transcendence values (β = −0.048, *t* = 1.676, *p* = 0.092), and civic attitudes (β = 0.049, *t* = 1.742, *p* = 0.082) on digital citizenship. The same is true for the Italian context, the analysis showed no significant mediating role of digital experiences from self-transcendence values (β = 0.002, *t* = 0.068, *p* = 0.946), and civic attitudes (β = 0.002, *t* = 0.111, *p* = 0.912). Table 5 shows mediational analysis results.

**Table 5:** The results of the mediation analysis.

Hypotheses	Romania				Italy				Complete			
	B	t	*p*-value	Results	B	t	*p*-value	Results	B	t	*p*-value	Results
H6:CA->DE->DC	−0.048	1.676	0.094	Not supported	0.001	0.068	0.946	Not supported	−0.035	2.020	0.044	Supported
H7: SE->DE->DC	0.049	1.742	0.082	Not supported	0.002	0.111	0.912	Not supported	0.034	2.105	0.036	Supported

*Note. B* = Beta Coefficient, *T* = *t* – Statistics, *P* = Probability (P) value.

### 4.4 Multigroup analysis

In the study's final phases, the cross-national comparisons of digital citizenship in the Romanian and Italian samples were performed, in terms of civic attitudes, digital experiences, and self-transcendence values. The findings suggested that the two areas with the greatest discrepancies are the influence of civic attitudes and self-transcendence values on digital experiences. The differences in route coefficients showed that civic attitudes and self-transcendence ideals on digital experiences are more influential in the Italian than in the Romanian sample. An overview of the outcomes of the multigroup study is given in Table 6.

**Table 6:** The results of the multigroup permutations analysis.

Relationships	Difference (Romania – Italy)	*p*-value
Hypotheses	−0.06	0.556
CA-> DC	0.209	0.031*
CA->DE	0.047	0.653
DE-> DC	−0.008	0.954
SE-> DC	−0.340	0.007*
SE->DE	−0.066	0.556

*Note:* CA: civic attitudes; DC: digital citizenship; SE: self-transcendence. *The Differences are significant in the relationships between the two countries (*p* < 0.05).

## Limitations

The dataset presented shows a few limitations, but this does not alter the significance of the results. First, the sample size is not large enough for the results to be representative for the entire population. While acknowledging the small sample size, we especially targeted university students for the study. Further, the Romanian sample was primarily constituted of females (96 %, *N* = 336) creating a gender imbalance. It should also be noted that the data used in this study is based on self-reported responses and this may represent another limitation source. As such, it is strongly encouraged that future research supplement this constraint by taking advantage of alternative techniques for data collection. However, the data is valuable for exploring cross-national variability.

## Ethics Statement

The research followed the guidelines of the Declaration of Helsinki and received the ethics approval no 1184/08.09.2023 from the ethics committee of Alexandru Ioan Cuza University. Informed consent of all participants has been obtained, and their data were fully anonymized. Participants were informed that their involvement was entirely voluntary, and they had the option to withdraw at any time. Detailed information was provided regarding the data security, the nature of the collected information, the data storage procedures, and the measures implemented to preserve participant anonymity. Respondents were informed that the data would only be used for academic purposes.

## CRediT Author Statement

**Popa Nicoleta Laura:** Conceptualization, Writing. **Assante Gabriela Monica:** Conceptualization, Methodology, Formal analysis, Writing.

## Data Availability

OSFData set on explanatory factors of digital citizenship among university students (Original data). OSFData set on explanatory factors of digital citizenship among university students (Original data).
